# Positive Factors for Return to Work After Accidents: Health Awareness, Consultation with Doctors, and Personal Characteristics of Workers

**DOI:** 10.1016/j.shaw.2021.10.001

**Published:** 2021-10-13

**Authors:** Dongsuk Kang

**Affiliations:** Department of Business Administration, College of Social Sciences, Gangneung-Wonju National University (GWNU), Street 7, Jukheon-gil, Gangneung-si, Gangwon-do, 25457, Republic of Korea

**Keywords:** Industrial management, Information service, Policy effect, Productivity, Vocational rehabilitation

## Abstract

****Background**:**

Industrial accidents can determine the overall level and quality of the work environment in industries and companies that contribute to national economic development. Korea has transformed the country from an international aid recipient to a donor country, but it has ranked first among the Organisation for Economic Co-operation and Development member countries in the number of fatal industrial accidents. Little has been known about the policy effects in terms of the workers' insurance for their industrial accidents and rehabilitation. This study raises two research questions about the influence of workers' personal characteristics and vocational rehabilitation services on their return to workplaces.

****Methods**:**

The study implements weighted logistic regression analysis using propensity score matching. This research utilizes the relevant dataset (3,924 persons) of Korea's industrial accident and insurance.

****Results**:**

The findings show that the level of workers' awareness of health recovery and their counseling for rehabilitation by physicians had positive effects on their return to work. Environmental factors such as workers' job stability at the time of industrial accidents and the temporal effects of industrial accidents (e.g., the level of disability, their age) had negative impacts on their return to work.

****Conclusions**:**

These findings have policy implications that the concentration of rehabilitation services for patients who have been mildly affected by industrial accidents would be effective in the short and medium term. The findings also highlight the necessity of ongoing policies about workers' vocational recovery with concrete evidence about policy impacts.

## 1 Introduction

### 1.1 Research background

Industrial accidents can symbolize the quality of the labor welfare and work environment of workers that would contribute to the economic development of a country. Among the Organisation for Economic Co-operation and Development (OECD) member countries, the Republic of Korea has achieved rapid economic growth as a country that has transformed from an international aid recipient to a donor country [1,2]. However, Korea ranked first among OECD member countries in the number of fatal industrial accidents (9.6 cases) compared to 100,000 workers in 2016, and the number of workers involved in industrial accidents has remained at 90,000 over the past 10 years [3].

**Fig. 1:**
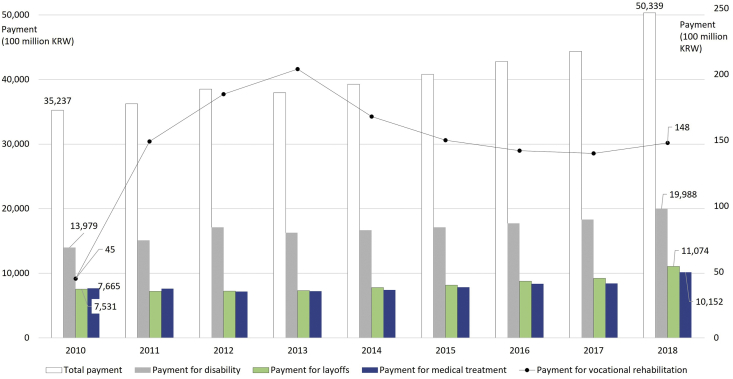
Statistics of the insurance payment for industrial accidents in Korea.Note. KRW: Korean Won. Source: MOEL [4], revised work by the author

The financial amount of industrial accident insurance payments has been increasing continuously (Fig. 1). The proportion of disability benefits has gradually increased from 35,237 billion Korean Won (KRW. 39.67%) in 2010 to 50,339 billion KRW (56.72%) in 2018 [4]. The share of vocational rehabilitation benefits about workers' vocational training and rehabilitation for re-employment has increased quantitatively from 4.5 billion KRW (0.13%) in 2010 to 14.8 billion KRW (0.42%) in 2018, but the proportion of the benefits is relatively low [4]. This low share of the benefits can suggest that vocational rehabilitation services for workers in industrial accidents need to improve their efficiency of performance due to their small budget sizes.

Since Korea's database of industrial accidents and insurance beneficiaries has changed and new additional information of workers (e.g., financial conditions) from 2018 has been incorporated [3,5], researchers need to reflect this informational innovation for more realistic analyses than in the past. Therefore, this study utilizes this recent dataset considering workers' awareness about their health recovery and other information (e.g., assets) and analyzes the various rehabilitation services' (e.g., counseling with the attending physician) employment effect on the job market as new research contributions.

This study raises the following **two research questions (RQs)** about the influence of workers' personal characteristics and vocational rehabilitation services on their return to their workplaces. **RQ1.** What features of workers influence their return to work? **RQ2.** What vocational rehabilitation services are effective for their employment?

This study utilized analysis of multivariate logistic regression and the weights of the propensity score method for a more exact analysis of the workers' groups by reflecting various rehabilitation services, such as their personal features related to their industrial accidents and counseling from their doctors. The results showed that the counseling service of the attending physician had a positive effect on workers returning to work. Furthermore, the perception of their health status and stability of the job status at the time of the industrial accident had an affirmative effect on their return to work. This study has public implications that governments need to strengthen policies of vocational rehabilitation for workers linked to their health improvement in the short to medium term.

### 1.2 Literature review on factors influencing workers' return to work after accidents

Workers' return to work due to the support of public insurance for industrial accidents could be relevant to the impact of vocational rehabilitation services and people's use of health-related services. The aspects of the workers' population, psychology, and the level of medical services for occupational accidents, rehabilitation-related activities are influencing variables that contribute to their returning to the labor market or the original workplace of people who encountered neck, back, and shoulder injuries at work [[6], [7], [8]].

For example, several demographic factors (e.g., gender, income) and psychosocial factors (e.g., a person's confidence) are important variables that can positively influence patients' return to their jobs. In addition, rehabilitation factors (e.g., completion of rehabilitation programs) are variables that affect their return to work [8].

Therefore, this study included rehabilitation activities (e.g., relevant services of the insurance for industrial accidents, information gains about the services), workers' experience (e.g., job stability [full-time or not], job classification [manager, laborer]), characteristics of the company (size, industry classification), and demographic characteristics (e.g., gender, marital status) as plausible variables positively affecting workers' return to work. Korea has unique geographic features where high-quality jobs are concentrated in areas of Seoul, Gyeonggi, or Incheon. This study used a dummy variable of workers' residency with regard to the area with the assumption of workers' physical proximity between their residential area and workplace.

In addition, the study adopted the variables of the severity of the accident damage to the workers, the resulting reduction in their work competency, and their job experiences before the accident [3]. The study included a variable of a task category on whether workers are managers or laborers; the labor market can be divided into two parts, namely a primary labor market, which can expect relatively high wages, high professionalism, and job position (e.g., managers), and a secondary labor market which can expect low wages, simple work, or temporary work [9].

In contrast, demographic aspects (e.g., age, debt, a period of sick leave), psychosocial aspects (e.g., depression, health concerns), and rehabilitation factors (e.g., unfavorable workplaces to support the insurances) are variables that can negatively affect workers' return to work [8]. If workers are in a favorable environment (e.g., youth, higher education, stable workers, high income, and marital status), they have a higher probability of returning to work [8].

Therefore, this study reflects the aspects of workers' positive perception in their health recovery after encountering an accident, their household income, and their level of education (i.e., bachelor's or higher degree) as variables expected to positively affect their return to work. This study also included their age, the level of their damages from industrial accidents, and the nature of the accidents (i.e., occupational diseases, accidental injury) as variables expected to negatively influence workers' return to work. Furthermore, the insurances and the rehabilitation services for industrial accidents in Korea currently consist of vocational rehabilitation (i.e., consultation with a doctor, evaluation of workers' vocational ability, vocational education) and social rehabilitation (i.e., psychological counseling), which provide relevant information and education to the beneficiaries or workers after the accident depending on their choice [3,5].

## 2 Materials and methods

### 2.1 Materials: Sample data

This study utilized the 2018 sample data from the panel study of workers' compensation insurance (PSWCI) provided by Korea Workers’ Compensation & Welfare Service (KCOMWEL). The data were gathered using a systematic survey method whereby well-trained interviewers checked answers (face-to-face) from insurance beneficiaries who encountered occupational accidents. The surveyed data were confirmed by KCOMWEL [3,5].

The 2018 survey data is the first sample of the second cohort survey and panel data that tracks and investigates workers who receive industrial accident insurance every 5 years [3,5]. The cohort survey newly investigated the information such as the state of industrial accidents, emergency treatment, working environment, job history, and the beneficiaries' households including assets [3,5].

The measurement unit of this study is a worker who had experienced industrial accidents and benefited from health and financial support from the Korean government's insurance for the accidents; their insurance support also ended in 2017 and they were surveyed in 2018 [3]. This study defined dependent variables, policy variables, and independent variables based on previous studies (the Subsection 1.2) and established the estimation model of (3), (4) (Table 1). This study defined the dependent and dummy variable as workers' employment status in terms of whether they had returned to their original paid job or transferred to another paid job (dummy(*D*) = 1) or unemployed (*D* = 0).

**Table 1:** Definitions and descriptive statistics of variables (observations: 3,294)

Category		Meaning	Scales	Details in measurements	Mean	SD
Dependent variable	The employed	Waged workers	Nominal	1: Waged workers (those returning to their original job or moving to a new job) 0: Others (Base)	—	—
Policy variables (independent variables)	Information services for vocational rehabilitation	A service of counseling with doctors	Nominal	1: Yes 0: No (Base)	—	—
		Evaluation of workers' ability	Nominal	1: Yes 0: No (Base)	—	—
		Vocational education or training after the accident	Nominal	1: Yes 0: No (Base)	—	—
		Workers' use of vocational rehabilitation services (total)	Nominal	1: Yes 0: No (Base)	—	—
		Workers' use of social rehabilitation services (total)	Nominal	1: Yes 0: No (Base)	—	—
	Preparation for vocational rehabilitation	Workers' information gain regarding rehabilitation services (number cases)	Ratio	10: Workers obtained information about all 10 rehabilitation services 9: Workers obtained information about nine services — 0: Workers did not obtain any information about the services	4.250	4.066
		Workers' licenses (cases)	Ratio	0 (None), 1 (a license), 2,…, 10	0.490	0.973
		Job experiences after the accident (cases)	Ratio	0 (a case), 1,…, 10 ※Removal of the job at the time of an industrial accident	1.676	1.326
Independent variables	Levels of workers' perception of heath recovery	Degrees of their thinking about health improvement after the accident	Ordinal	1 (No recovery at all), 2 (Not recovered fully yet), 3 (undergoing recovery), 4 (Somewhat recovered), 5 (Fully recovered)	2.674	1.089
	Demographic factors	Age	Ordinal	4 (Age 60 or older), 3 (Age 50), 2 (40), 1 (Age 30 or younger)	2.736	1.032
		Gender	Nominal	1: Male 0: Female (Base)	—	—
		Marital status (at the time of the accident)	Nominal	1: Married 0: Others (Base)	—	—
		Levels of education	Nominal	1: Bachelor's degree or higher 0: Others (Base)	—	—
		Household income (10,000 Korean Won) ※Values of the natural log	Ratio	Total income level of workers' household in 2017 including labor, business, and other incomes	10.718	0.648
		Residential areas	Nominal	1: Metropolitan areas of Seoul, Incheon, Gyeonggi, and neighboring regions 0: Others (Base)	—	—
	Features of the accidents	Types of accidents	Nominal	1: Diseases or accidents occurring at workplaces 0: Accidents caused by commuting activities (Base)	—	—
		Levels of workers' disability	Ordinal	14 (1st grade), 13 (2nd grade),…, 1 (14th grade), 0 (No disability)	3.099	3.154
	Characteristics of the workplaces	Company size (Number of employees)	Ordinal	8 (More than 1,000 employees), 7 (300-990), 6 (100-299), 5 (30-99), 4 (20-29), 3 (10-19), 2 (5-9), 1 (less than 5 employees)	3.398	1.979
		Industry groups	Nominal	Manufacturing (dummy 01), Construction (dummy 02), and Others (Base)	—	—
		Managerial jobs	Nominal	1: Professional or managerial jobs (Manager, experts, office workers, machine controller, skilled worker in agriculture, forestry and fishery) 0: Others (Base)	—	—
		Job stability	Ordinal	3 (Full-time worker), 2 (Temporary workers), 1 (Daily workers), 0 (Others)	2.223	0.929

—, Not available because of a variable's feature (e.g., a dummy variable); Base, Base or reference group in each dummy variable; SD, Standard deviation.

This study adopted five representative services: workers' consultation with attending physicians, evaluation services about their work ability, vocational education, vocational rehabilitation services (i.e., total number of their use), and social rehabilitation services (i.e., total number of their use) [3]. The study also reflected the level of workers' awareness of health recovery and their demographic information at the time of the accident, including their age, dummy variables of gender, marital status, bachelor's degree or higher, residential location in Seoul or a metropolitan area, and their household income. The income was calculated as the values of natural logarithms in order to control the income variable's heteroscedasticity [10].

The study included the level of the workers' industrial accident, characteristics of their workplace at the time of the accident (i.e., company size and its industry group), occupational characteristics at the time of the accident [9], and their job stability as full-time workers [9,11,12] as control variables.

The descriptive statistics (Table 1) show that the total number of units of the sample data was 3,294, and there seems to be no missing values for 22 variables. This study checked the correlation and variance inflation factor (VIF) of variables to secure the statistical significance of model estimation; the maximum value of the correlation between the dependent and independent variables was 0.3. For example, the range of the VIF value is [1, 1.38], which seems to have no serious multicollinearity among variables because the relevant values were lower than 5 [13].

### 2.2 Methodologies: Propensity score matching and weighted logistic regression with propensity score matching

**Fig. 2:**
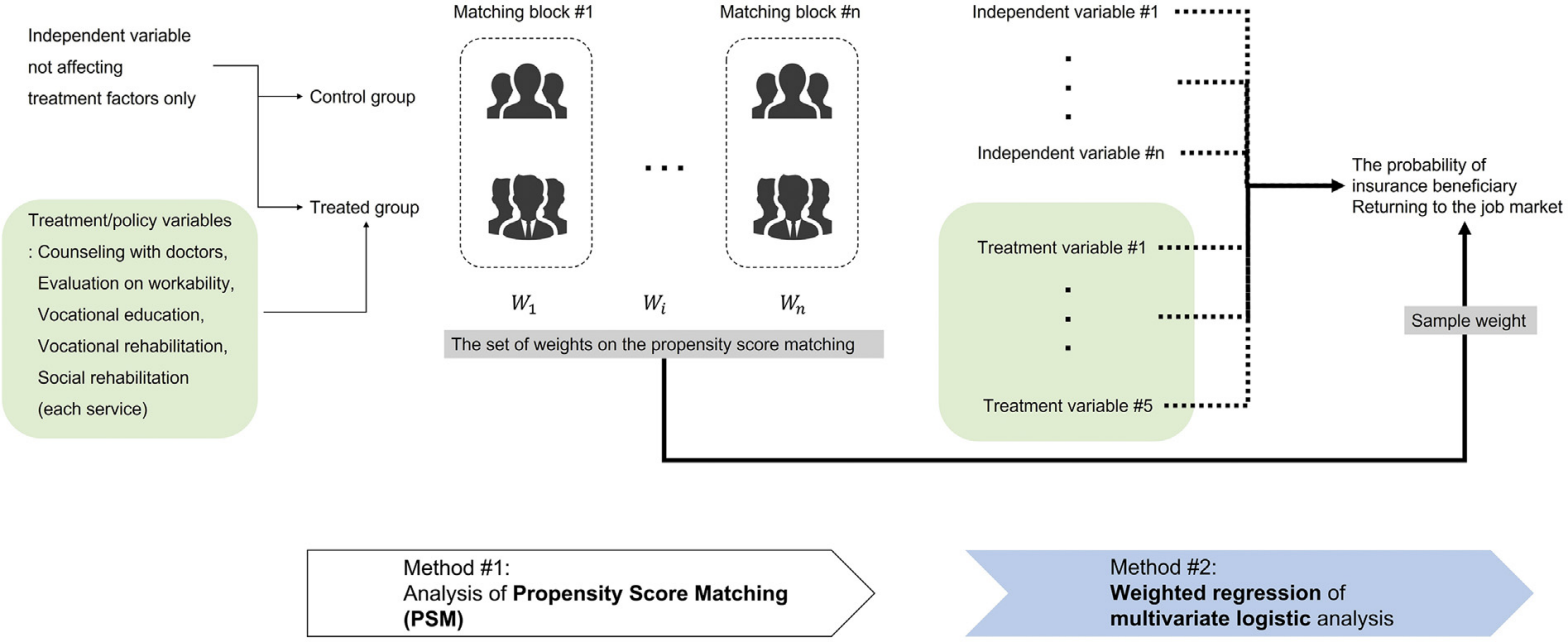
The two-stage analysis of this study: PSM and weighted regression of multivariate logistic analysis.

This study adopted the two-stage analysis of propensity score matching (PSM) and weighted logistic regression analysis using the weights of propensity scores to rigorously investigate the influence of key variables on workers' return to their work (Fig. 2). PSM analysis is one of representative methodologies that can statistically identify and compare the treated/target group and the untreated/control group using a policy variable in order to minimize biases caused by non-random sampling in non-experimental or observational data [14,15]. It estimates the conditional probability of other observable control variables for the policy variable through logistic regression, and it matches the treated group affected by the policy variable and the control group with the same conditions as long as possible.

To perform PSM analysis, it is important to select the appropriate control variables for the matching of the untreated group, and the variables should be the factors that affect both the policy variable and the dependent variable or those that affect only the dependent variable [16]. Researchers should offer sufficient evidence for the (un)selection of the control variable on how they are observed or time-invariant before the policy variables are measured, which should be based on previous studies or relevant theories [14].

Therefore, this study estimated PSM for each of the five policy variables. The study included control variables and excluded the dependent and other policy variables in order to estimate the control groups. For example, 10 control variables were selected, which were measured before benefiting from the accident insurance or were time-invariant: the classification of the accidents, the level of disability, workers' firm size at the time of the accident, the job classification of workers at the time (a manager or not), their occupational positions, gender, marital status, level of education, and their household income.

Researchers should check the fulfillment of two conditions (i.e., unconfoundedness and common support) in order to perform PSM analysis for the match between the target group and the control group. First, unconfoundedness (or conditional independence assumption, selection of observables) is a strong condition that (possible) estimated outcomes (conditional on observable control variables that are not affected by policy or treatment variables) that must be independent of policy variables (Eq. 1) [14].

Next, common support (or overlap) means that the subjects with a policy variable of 1 (or treated) should have a probability value between 0 and 1, which is the probability conditional on its control variable; the subjects with the same value of control variables should have probabilities that are not all zero (Eq. 2**)** [14]. When these two conditions are satisfied; the PSM estimator for the average treatment effect on the treated group (ATT) is Eq. (3) [14].

(1) Y(0),Y(1)⊥T|X

(2) 0<Pr(T=1|X)<1

(3) $$M_{ATT}^{PSM}=E_{\left(\mathrm{Pr}\left(X\right)|T=1\right)}\left\{\left[Y\left(1\right)|T=1,\mathrm{Pr}\left(X\right)\right]-E\left[Y\left(0\right)|T=0,\mathrm{Pr}\left(X\right)\right]\right\}$$

This research establishes (1), (2), (3) which are the revised version of mathematical notations that Caliendo and Kopeinig [14] expressed. *T*, a policy variable (*T* = 1: the variable is applied; *T* = 0: the variable is not applied); $Y_{i}$ (*i* = 1, 2,…, *n*), plausible outcomes; *X*, all control variables that are not dependent or policy variables; *Pr(*·*)*, a value of a probability; *E(*·*)*, an expected value; $M_{ATT}^{PSM}:$, an estimation methodology of PSM about ATT.

This study performed the analysis step for every five policy variables and adopted kernel matching (conditions: Epanechnikov kernel and bandwidth of 0.06 [15]) from several representative matching algorithms. The study utilized the Stata program to perform the above two-stage analysis and the Stata packages of pscore [14,15,17] and psmatch2 [14,15,18,19] for the PSM analysis.

As the last step in the two-stage analysis, this study implemented the logistic model on the relationship between workers' influential variables and the outcomes on their return to work ((4), (5)) [13,20,21]. The study performed the weighted logistic regression, which utilizes the weights of propensity score derived from the PSM analysis using the sample data of 2018.

(4) Pi=Pr(Yi|Xi)=11+e−(β0Wi+βiWiXi).

(5) Li=ln(Pi1−Pi)=β0Wi+βiWiXi+ui.

**Fig. 3:**
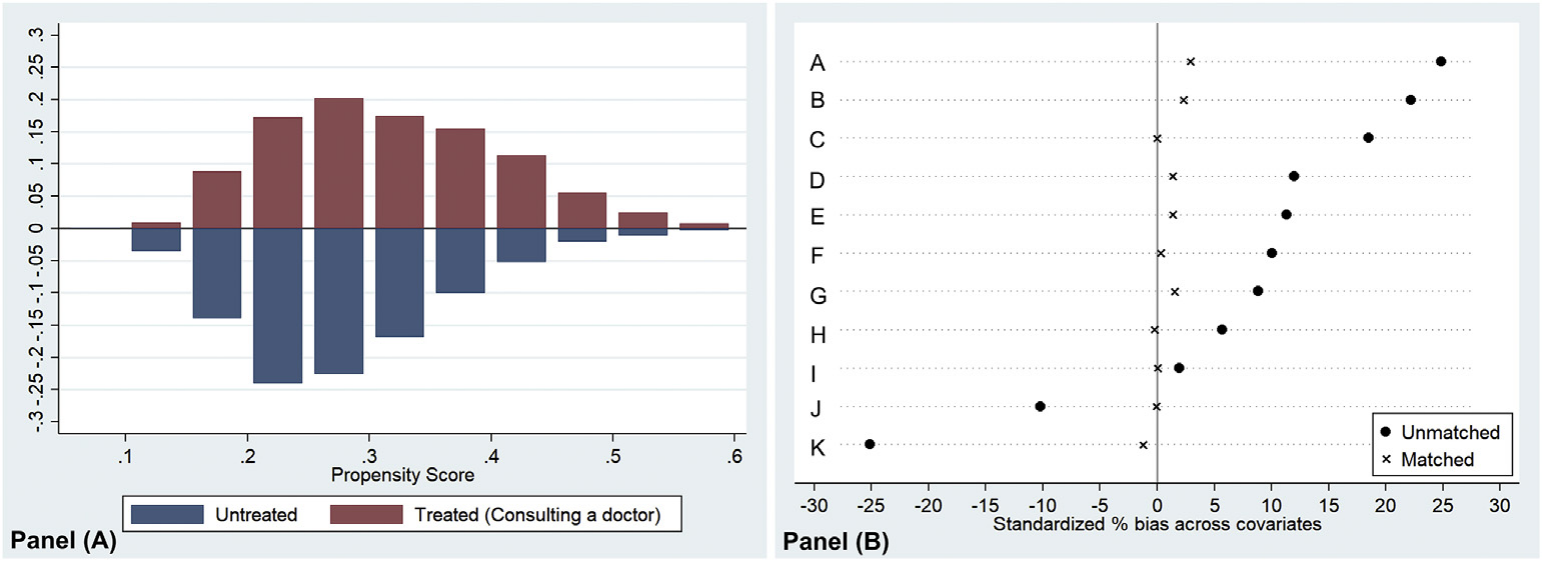
Comparison of the distribution of disposition scores and the convenience of control variables between the two groups, focusing on the counseling policy variables of the attending physician. ∗Notes. A: Company size. B: Job stability. C: Bachelor’s degree. D: Managerial Job. E: Household income. F: Residency in a metropolitan area. G: Marital status. H: Gender. I: Type of accident. J: Level of disability. K: Age.

This research establishes Eqs. (4), (5) which are the revised version of mathematical notations that Gujarati and Porter [13] and Rodrigues et al. [21] expressed. $Y_{i}$, a dependent variable; $\beta_{0}$, a constant term; $\beta_{i}$, coefficients of variables $X_{i}$; $W_{i}$, a propensity score weights calculated using Eqs. (1), (2), (3) and Fig. 3; $X_{i}$, independent variables including policy variables; $u_{i}$, a residual term, *i* = 1, 2,…, *n*.

## 3 Results

This study performed analyses of PSM and weighted logit regression for five policy variables of rehabilitation services. As a representative example of the results, this study presented the results table on the effects of the counseling variable. The graph analysis of the distribution status of propensity scores for the two groups also showed a similarity between the groups for the reasonable group comparison (Fig. 3). The biases of the control variables between the two groups were closer to zero than before the connection (Fig. 3). This research also implemented *t*-test about the difference between the groups [14,15] and the result was insignificance of the group differences.

As the main result, Table 2 shows the marginal effects of PSM and weighted logit analyses for five policy variables of rehabilitation services. In general, worker’s health recovery and counseling with a doctor had positive influences on their return to work. Job stability, household income, company size, and residency in a metropolitan area had positive influences on the return to work. However, workers' features, their level of disability, and their age had negative impacts on return to work. Policies of various rehabilitation services had partially influenced workers' return to work due to their policy differences. Other demographic factors also had limited impacts on workers' return to work.

**Table 2:** Result of marginal effects of five policy variables: Logit regression before PSM and weighted logit regression after PSM

Independent variables	Before PSM	After PSM about each of five policy variables (the row below)				
		Counseling with a doctor	Evaluation about workability	Vocational education	Service of vocational rehabilitation	Service of social rehabilitation
Counseling with a doctor (*D*)	0.0749∗∗∗	0.0726∗∗∗	0.0626∗	0.0521	0.111∗∗∗	0.102∗∗∗
	(0.0232)	(0.0209)	(0.0324)	(0.0670)	(0.0414)	(0.0323)
Evaluation of workability (*D*)	0.0600∗	0.0503	0.0523	0.166∗	−0.00393	0.0823∗
	(0.0339)	(0.0328)	(0.0318)	(0.0940)	(0.0582)	(0.0477)
Vocational education (*D*)	−0.170∗∗∗	−0.166∗∗∗	−0.0761	−0.161∗∗	−0.203∗∗	−0.150∗∗
	(0.0560)	(0.0576)	(0.0748)	(0.0644)	(0.0829)	(0.0760)
Vocational rehabilitation (*D*)	−0.0598∗	−0.0442	−0.124∗∗∗	−0.142∗	−0.0587∗	−0.0410
	(0.0328)	(0.0338)	(0.0464)	(0.0821)	(0.0355)	(0.0443)
Social rehabilitation (*D*)	−0.00213	0.0165	0.0122	0.0592	0.0184	−0.00307
	(0.0255)	(0.0263)	(0.0362)	(0.0694)	(0.0445)	(0.0273)
Information gained about rehabilitation services	0.00212	0.000247	0.00382	0.00735	0.00496	0.00314
	(0.00246)	(0.00254)	(0.00347)	(0.00705)	(0.00423)	(0.00337)
Licenses	0.0248∗∗	0.0151	0.0150	0.0211	0.0326	0.0429∗∗∗
	(0.0118)	(0.0117)	(0.0154)	(0.0235)	(0.0224)	(0.0164)
Job experiences	0.0194∗∗∗	0.0188∗∗	0.0174	−0.0208	0.0111	0.00596
	(0.00749)	(0.00797)	(0.0112)	(0.0204)	(0.0121)	(0.0102)
Health recovery	0.106∗∗∗	0.0989∗∗∗	0.0957∗∗∗	0.0706∗∗∗	0.0880∗∗∗	0.112∗∗∗
	(0.0101)	(0.0104)	(0.0141)	(0.0260)	(0.0163)	(0.0139)
Type of accident (*D*)	0.0882	0.0358	−0.0504	0.0958	0.239∗	0.0257
	(0.104)	(0.0936)	(0.112)	(0.119)	(0.144)	(0.105)
Level of disability	−0.0388∗∗∗	−0.0318∗∗∗	−0.0305∗∗∗	−0.0353∗∗∗	−0.0401∗∗∗	−0.0442∗∗∗
	(0.00341)	(0.00363)	(0.00478)	(0.00978)	(0.00619)	(0.00465)
Age	−0.0635∗∗∗	−0.0551∗∗∗	−0.0526∗∗∗	−0.0185	−0.0563∗∗∗	−0.0510∗∗∗
	(0.0109)	(0.0112)	(0.0155)	(0.0298)	(0.0185)	(0.0150)
Gender: Male (*D*)	0.0490∗	0.0568∗	0.00463	0.0735	0.0671	0.0974∗∗
	(0.0284)	(0.0297)	(0.0434)	(0.0710)	(0.0489)	(0.0386)
Marital status: Marriage (*D*)	0.0591∗∗∗	0.0609∗∗∗	0.0269	0.0946	0.115∗∗∗	0.0785∗∗
	(0.0218)	(0.0228)	(0.0320)	(0.0676)	(0.0394)	(0.0316)
Bachelor's degree (*D*)	0.0148	0.0231	0.0268	−0.0508	0.00252	0.0188
	(0.0288)	(0.0277)	(0.0393)	(0.0769)	(0.0489)	(0.0401)
Household income	0.0841∗∗∗	0.0883∗∗∗	0.107∗∗∗	0.0147	0.0747∗∗	0.100∗∗∗
	(0.0168)	(0.0172)	(0.0243)	(0.0457)	(0.0294)	(0.0262)
Residency in a metropolitan area (*D*)	0.0635∗∗∗	0.0389∗∗	0.0475∗	0.0220	0.105∗∗∗	0.116∗∗∗
	(0.0193)	(0.0198)	(0.0275)	(0.0522)	(0.0334)	(0.0266)
Managerial job (*D*)	0.0306	0.0252	−0.00687	0.0597	−0.00728	0.0189
	(0.0241)	(0.0246)	(0.0343)	(0.0680)	(0.0412)	(0.0345)
Job stability	0.0564∗∗∗	0.0571∗∗∗	0.0640∗∗∗	0.0779∗∗	0.0682∗∗∗	0.0742∗∗∗
	(0.0133)	(0.0141)	(0.0198)	(0.0372)	(0.0224)	(0.0186)
Company size	0.0159∗∗∗	0.0172∗∗∗	0.0118∗	0.0255∗	0.0175∗∗	0.0107
	(0.00512)	(0.00516)	(0.00700)	(0.0144)	(0.00862)	(0.00715)
Manufacturing industry (*D*)	0.0470∗	0.0254	0.0799∗∗	0.0313	−0.00456	0.0122
	(0.0248)	(0.0253)	(0.0350)	(0.0728)	(0.0434)	(0.0342)
Construction industry (*D*)	0.0198	0.00625	0.00168	0.0625	−0.0174	−0.0242
	(0.0288)	(0.0303)	(0.0431)	(0.0780)	(0.0494)	(0.0402)
Observations	3,292	3,292	3,292	3,292	3,292	3,292
Wald chi-square test (DF: 22)	549.73∗∗∗	405.13∗∗∗	251.30∗∗∗	73.70∗∗∗	197.49∗∗∗	385.19∗∗∗

Robust standard errors in parentheses. All predictors at their mean values.

D, a dummy variable; DF, degree of freedom.

∗∗∗ *p* < 0.01, ∗∗ *p* < 0.05, ∗ *p* < 0.1. All predictors at their mean values.

## 4 Discussion

This study analyzed the influence of vocational rehabilitation services on workers returning to work after industrial accidents. To answer **RQ1** (the employment effect of workers' features), the level of workers' awareness of health recovery from an accident, job stability at the time of the accident, household income, and residency in a metropolitan area had positive contributing effects on their return to work (Table 2). Relevant studies confirm the positive influences of their psychological factors, participation in rehabilitation activities, and the location of residency [[6], [7], [8]]. In addition, the level of workers' disability caused by accidents and their age had a negative influence on their overall return to work; these results were relatively low compared to those derived from the analysis before PSM. Previous studies have also confirmed the negative effects of demographic factors [[6], [7], [8]].

Next, to answer **RQ2** (the effect of rehabilitation services on workers' return to work), among several policy variables, the “counseling service with a doctor” variable had a positive influence on the workers' return, and this influence changed slightly in comparison with that established from the analysis before PSM. The “evaluation of workers' ability” variable seems to have positive effects in some of the results.

These findings suggest two possible implications for improving the performance of the public policies of rehabilitation services for workers encountering industrial/vocational accidents. Above all, workers' health recovery and their counseling from doctors have a positive influence on their returning to the job market (Table 2). It could be important to focus more on rehabilitation services about their health and vocational recovery in the short and medium term. For example, if workers actively participate in rehabilitation services of doctors' counsel, those who have positive attitudes toward their daily lives and health seem to have a higher potential for work than those who do not. On this point, a government needs to concentrate on supporting policies for workers with active minds to allow them to work and gain much experience in the services sector so as to improve policy efficiency and effectiveness.

Furthermore, the effects of the workers' economic and social environment (e.g., job stability, household income) at the time of the accidents and the temporal effects of the accident (e.g., disability level) can influence their return to work (Table 2). If laborers have a somewhat good professional career (e.g., a full-time job, a high wage) and have experienced mild levels of accidents, they could be favorable targets of a government's rehabilitation policies. On the other hand, workers who have experienced part-time jobs and suffer from severe disabilities caused by their accidents seem to have a low potential for work and seem to be ineffective policy consumers due to their unfavorable conditions. Therefore, a government should design its policies targeting differences in the workers' environment in accordance with their work cycle or the timing of their accident and recovery.

## 5 Conclusions

This study performed PSM and multivariate logit analysis to evaluate the effects of workers' various characteristics and policies of rehabilitation services on employment. The results of the several analyses showed that workers' perception of health recovery and health counseling services by their doctors had positive effects on their return to the job market. In addition, environmental factors before industrial accidents and temporal effects of industrial accidents (e.g., disability level, age) had negative effects on their return. These findings suggest that vocational rehabilitation policies directly connected to health recovery in the short and medium term will be effective for workers' predictable return to their work considering governments' limited policy resources. Future research needs to investigate the long-term effects of industrial accident insurance using panel regression analysis on multi-period datasets. For example, future studies could analyze the factors influencing employment for workers encountering occupational accidents and benefiting from insurance for more than a year.

## Conflicts of interests

The author has no conflicts of interests to declare.
